# Rituximab in rheumatology: single-centre SARS-CoV-2 infection and COVID-19 prevalence

**DOI:** 10.1093/rap/rkac009

**Published:** 2022-03-10

**Authors:** Elliott Lever, Matthew Colquhoun, Kalveer Flora, Shawki El-Ghazali, Rohit Baslas, Yasir Suleman, Amy Wieckowski, Ziad Farah, Shahir Hamdulay, Anthony Isaacs, Jaita Mukherjee, Francis Pazos, Henry Penn, Pedro M Machado

**Affiliations:** 1 Department of Rheumatology, Northwick Park Hospital, London North West University Healthcare NHS Trust; 2 National Institute for Health Research (NIHR) University College London Hospitals (UCLH) Biomedical Research Centre (BRC), UCLH NHS Foundation Trust; 3 Centre for Rheumatology, Division of Medicine; 4 Department of Neuromuscular Diseases, UCL Queen Square Institute of Neurology, University College London, London, UK

Key message

- We describe a coronavirus disease 2019 (COVID-19) prevalence of 20% in rheumatic disease patients treated with rituximab, with 10.3% of these (2% of the entire cohort) having died of COVID-19-related causes.

Dear Editor, Observational registry data have been published to date suggesting an increased risk of severe coronavirus disease 2019 (COVID-19) in rheumatic disease patients treated with rituximab [1–4]. Evaluation of risk associated with immunosuppressive or immunomodulatory medication is essential for the management of rheumatic and other immune-mediated or autoimmune conditions.

At a busy district general hospital in London, we looked at all rheumatology patients who had received rituximab between September 2018 and February 2021 and reviewed their electronic hospital record or contacted the patient by telephone. Verbal consent was obtained for patients who were contacted by telephone. Patients were asked if they had tested positive for severe acute respiratory syndrome coronavirus 2 (SARS-CoV-2) before April 2021; had symptoms of COVID-19 but tested negative or did not have a test; and how strictly they had followed UK government guidelines to shield or stay at home (rating from one to five, on a Likert-type scale, with higher scores representing more strict adherence to government guidelines).

During the study period, 148 patients received rituximab and 34 (23%) received more than one cycle. The mean age of the case series was 60 years (SD 14.5 years), and 85% were female. The most frequent diagnosis was RA (70%), with the remaining diagnoses being vasculitis (7.5%), SLE (5%), other CTD (14%) and IgG4-related disease (4%). The ethnicity of the patients included 32% Asian Indian, 32% any white background and 14% any black background; the remainder (22%) were of other ethnic background. Patients on synthetic DMARDs at the time of the study included: HCQ 32%, SSZ 13.5%, MTX 43%, LEF 3%, AZA 3%, MMF 11% and glucocorticoids 39%.

Five patients were not contactable by telephone and excluded from the analyses (*n* = 143). The incidence of SARS-CoV-2 PCR-proven infection was 15 of 143 (10.4%), and 14 of 143 (9.8%) reported COVID-19 symptoms but tested negative, resulting in a total of 29 of 143 (20.3%) with definite/probable COVID-19 illness, half of them reporting moderate or high rheumatic disease activity.

COVID-19-related death occurred in 3 of 143 (2%) patients (3/29 = 10.3% of those with definite/probable COVID-19 illness), with 5 of 143 (3.5%) having died from non-COVID-19 causes (pneumonia and pulmonary fibrosis). Hospitalization occurred in an additional 2 of 143 (1.4%) patients (2/29 = 6.9% of those with definite/probable COVID-19 illness); these two patients survived, not requiring intensive care or high-dependency units. The average reported shielding rating was 4.7/5. The incidence of two or more co-morbidities was 28%, three or more 9%, and four or more 3%.

A short description of the three patients who died of COVID-19 is provided in Table 1.

**Table 1:** Summary of the three patients treated with rituximab who died of coronavirus disease 2019

Case	Age (years)	Sex	Co-morbidities	Concurrent medications	Date of last rituximab infusion	Date of hospitalization with coronavirus disease 2019
1	79	F	RA	MTX	December 2019	December 2020
			Left upper lobe lobectomy for adenocarcinoma	SSZ		
			Hypertension	No concurrent CSs		
			Ex-smoker, 60-year pack history			
2	73	M	RA	MTX	October 2020	January 2021
			Interstitial lung disease	Prednisolone, 5 mg daily		
3	59	M	Granulomatosis with polyangiitis, respiratory disease	MTX	September 2018	April 2020
			CMV viraemia	Prednisolone, 5 mg daily		
				CYC, 2018		

We extracted data from coronavirus.data.gov.uk and ons.gov.uk for the three local boroughs, Harrow, Brent and Ealing, population 0.93 million. For the study period, COVID-19 prevalence was 7.6% with a mortality of 3.3%; both were higher in our patient cohort, suggesting the vulnerability of this patient group owing to co-morbidity, age and, potentially, exposure to rituximab treatment.

The data provide valuable perspective on the risk of severe COVID-19 or death in a collection of patients with rheumatic disease treated with rituximab and with multiple co-morbidities. In an international registry, Strangfeld *et al.* [1] reported an association between COVID-19-related death and rituximab (compared with MTX monotherapy); 42 of 193 (21.8%) COVID-19 rheumatic disease patients treated with rituximab died. Mortality for rituximab monotherapy compared with combination therapy was 25 of 91 (27.4%) and 17 of 102 (16.7%), respectively [1]. In our patients, 20.3% acquired COVID illness, and among these the mortality was 10.3%; however, the data are not directly comparable. A Swedish national data set also identified increased risk of COVID-19-related death with rituximab and Janus kinase inhibitors (compared with csDMARDs) [3]. More patients on rituximab died of other causes during the pandemic period. We have demonstrated that patients reported high adherence to UK government guidelines to shield or stay at home, but we can also identify that these patients still acquired COVID-19. Our data highlight the inherent difficulties in interpreting the data from small datasets of rituximab-treated patients because they are often multi-morbid, on other disease-modifying treatment in addition to previous rituximab and potential long-term immunoparesis. Although the data presented here help to frame the risk of COVID-19 to patients, in particular given that vaccine efficiency is diminished in rituximab-treated patients, only nationwide datasets will allow the avoidance of selection bias and the generation of findings that are generalizable across all age groups, ethnicities, geographical locations and socioeconomic, health and personal characteristics.

*Funding:* No specific funding was received from any funding bodies in the public, commercial or not-for-profit sectors to carry out the work described in this manuscript.

*Disclosure statement*: P.M.M. has received consulting/speaker’s fees from Abbvie, BMS, Celgene, Eli Lilly, Galapagos, Janssen, MSD, Novartis, Orphazyme, Pfizer, Roche and UCB, all unrelated to this manuscript, and is supported by the National Institute for Health Research (NIHR), University College London Hospitals (UCLH), Biomedical Research Centre (BRC). M.C. received support to attend a conference from Pfizer. No other competing interests by the other authors.

*Ethics:* The study was approved as a service quality evaluation by the Research and Development Department at Northwick Park Hospital.

## Data availability statement

Data are available upon reasonable request by any qualified researchers who engage in rigorous, independent scientific research, and will be provided following review and approval of a research proposal and Statistical Analysis Plan (SAP) and execution of a Data Sharing Agreement (DSA). All data relevant to the study are included in the article.
